# Running in mice increases the expression of brain hemoglobin-related genes interacting with the GH/IGF-1 system

**DOI:** 10.1038/s41598-024-77489-1

**Published:** 2024-10-26

**Authors:** Marion Walser, Lars Karlsson, Reza Motalleb, Jörgen Isgaard, H. Georg Kuhn, Johan Svensson, N. David Åberg

**Affiliations:** 1 Department of Internal Medicine and Clinical Nutrition, Institute of Medicine, The Sahlgrenska Academy, University of Gothenburg, Gothenburg, Sweden; 2 Region Västra Götaland, Department of Clinical Chemistry, Sahlgrenska University Hospital, Laboratory of Experimental Endocrinology, Bruna Stråket 16, 413 45 , Gothenburg, Sweden; 3 Department of Neurology and Neurological Sciences, Stanford University, Stanford, CA USA; 4 Department of Clinical Neuroscience, Institute of Neuroscience and Physiology, The Sahlgrenska Academy at University of Gothenburg, Gothenburg, Sweden; 5 Region Västra Götaland, Department of Specialist Medicine, Sahlgrenska University Hospital, Gothenburg, Sweden; 6 Institute for Public Health, Charité – Universitätsmedizin Berlin, Berlin, Germany; 7 Region Västra Götaland, Department of Acute Medicine and Geriatrics, Sahlgrenska University Hospital, Gothenburg, Sweden

**Keywords:** Brain, Rodent, Exercise, Sedentary, Insulin resistance, Insulin-like growth factor-1, Neuroscience, Biomarkers, Endocrinology, Medical research

## Abstract

The beneficial effects of exercise are partly mediated via local or systemic functions of the insulin-like growth factor-1 (IGF-1) system. As IGF-1 increases local brain hemoglobin beta (Hbb) transcripts, we hypothesized that exercise could have similar effects. Mice were single-housed with free access to running wheels for seven days. After sacrifice and saline perfusion, the expression of 13 genes was quantified using real-time quantitative polymerase chain reaction (RT-qPCR) in three brain regions: the prefrontal cortex, motor cortex, and hippocampus. In addition, plasma insulin, glucose, homeostatic model assessment of IR (HOMA-IR), C-peptide, and IGF-1 were investigated. We show that hemoglobin-related transcripts (Hbb and 5’-aminolevulinate synthase 2 [Alas2]) increased 46–63% in the running group, while IGF-1-related genes [Igf1 / growth hormone receptor (Ghr)] decreased slightly (7%). There were also moderate to large correlations between Hbb- and IGF-1-related genes in the running group but not in the sedentary group. HOMA-IR, plasma glucose, and insulin changed marginally and non-significantly, but there was a trend toward an increase in plasma-IGF-1 in the running group. In conclusion, seven days of running increased Hbb-related transcripts in three brain regions. Hbb-related transcripts correlated with components of the brain IGF-1 system only in the running group.

**Supplementary Information:**

The online version contains supplementary material available at 10.1038/s41598-024-77489-1.

**Authors**:

## Introduction

It is widely recognized that endurance exercise improves mental well-being^1^, increases cerebral blood flow^2^, alters neurotransmitter release^3^, and modulates the growth factors insulin-like growth factor-1 (IGF-1) and brain-derived neurotrophic factor (BDNF)^4–6^. Exercise also improves neuroprotective resilience to ischemia^7^ and recovery after stroke^8^. During exercise, growth hormone (GH) is released^9^, and exercise also has effects of a similar type as GH administration on the brain and neuroprotection in hypophysectomiced (Hx) rodents^10,11^. For example, exercise increases neural progenitor cell proliferation, neurogenesis, and cognitive function^12,13^, quite similar to the effects of GH and IGF-1 administration^14,15^.

Circulating GH crosses the blood-brain barrier^16^ and binds to GH receptors expressed in the brain^17^. GH stimulates IGF-1 expression in the liver^18^ and, to a lesser extent, in other organs, including the brain^19,20^. IGF-1 is actively transported over the blood-brain barrier via carrier-mediated uptake^21,22^. Some of the effects of GH in the brain are thought to be mediated by endocrine IGF-1^23–25^. Indeed, the presence of both IGF-1 and IGF-1 receptors (IGF-1R) in neurons, glial, and endothelial cells^26,27^ enables endocrine and paracrine local effects of IGF-1 ^28–30^.

Hemoglobin beta (Hbb) is expressed in neurons^31,32^, specifically in dopaminergic neurons, and to some extent in mature oligodendrocytes and cortical and hippocampal astrocytes^33^. Brain Hbb is thought to have biological functions similar to those in erythrocytes^34^, acting as a functional oxygen reservoir^33^. Several precursors, including Hbb and heme, form hemoglobin. The oxygen-binding heme group is synthesized through a pathway whereby Alas2 catalyzes the first step by converting glycine and succinyl-CoA into 5-aminolevulinic acid (ALA)^35^. ALA is then further processed in several steps, the final product being heme, which is united with two hemoglobin alpha and two hemoglobin beta to a functional tetrameric hemoglobin^35,36^. The enzyme Alox 15 is expressed in the mouse brain and is important for memory function^37^. Specifically, Alox15 acts via oxidizing polyunsaturated fatty acids to generate bioactive lipid metabolites such as eicosanoids and lipoxins and has a role in oxidative and inflammatory reactions^38,39^. Alox15 probably also has another functional link with hemoglobin, as suggested by results from an Alox15b knock-in mouse, which has lower erythrocyte counts, hematocrit, and hemoglobin, possibly mediated by cell membrane instability and increased hemolysis^40^. Altogether, Hbb, Alas2 and Alox15 can be considered as being functionally related to oxygen metabolism. Notably, Alox15 is increased in the hippocampus in response to GH treatment^25^. Previously, we have shown that GH administration significantly increased the production of *Hbb*, *Alas2* and *Alox15* transcripts in the rodent brain^41,42^. Also, in the GH/IGF-1 deficient Lewis dwarf (dw/dw) rat, GH administration increased hemoglobin in the brain^25^. Considering that GH or IGF-1 administration has many similarities to exercise-induced effects in the brain, we hypothesized that exercise would also increase Hbb-like transcripts in the brain.

The present study aimed to investigate whether seven days of running exercise affects Hbb-related transcripts previously shown to be affected by GH/IGF-1, and whether there is evidence that the IGF-1 system mediates the effects seen. In mice subjected to voluntary running for one week, we analyzed three different parts of the brain, the prefrontal cortex, the motor cortex, and the hippocampus, all of which are involved in exercise^43–45^. We assessed 13 gene transcripts, further grouped into four categories: Hbb-like, neuron-, glia-, and IGF-1-related. Finally, we analyzed plasma glucose, insulin, C-peptide, and IGF-1 as exercise improves these markers of glucose metabolism^46^.

## Method

### Animals and running procedures

We used 24 six-month-old male mice of the strain C57BL/6J (Charles River, Germany) kept under standard temperature conditions (24 °C) and 50–60% relative humidity. The sample size (*n* = 12 in each group) was determined for allowing detection of a transcript 40% difference provided a 30% coefficient of variation, with 90% power and 5% significance level. A 12-h light/dark cycle was maintained with light from 19:00 to 07:00 (reversed) and *ad libitum* access to food and water.

Running wheel experiments were performed as previously described^47^. Mice were housed individually in cages with free access to low-profile running wheels (ENV-047, Med Associates, Fairfax, VT, USA). After one week of acclimatization, half of the running wheels were unlocked (i.e., animals were randomized to voluntary running or sedentary controls), allowing simultaneous access for the animals in the running group. Running wheel activity was wirelessly recorded throughout the experiment.

Mice in the running group could run at their discretion, typically during their active dark phase, while the wheels for the sedentary group remained locked, preventing any running activity. Running activity was continuously monitored using a telemetry system integrated with the wheels, ensuring accurate tracking of distance and time spent running. This data was used to assess each animal’s level of voluntary activity. Since the running wheels for sedentary animals were locked and could not rotate, their activity was not recorded. Other confounders were not controlled for, and analysis was not blinded. Animals were sacrificed after one week of running during their active phase in the dark cycle. No specific criteria for inclusions or exclusions were used other than healthy appearance (other specific humane endpoints not used). After sacrifice some laboratory analysis was not successful, but the numbers of included animals are reported in the tables.

At the time of euthanasia, mice were deeply anaesthetized with a peritoneal injection of 50 mg/kg sodium thiopental during the animals’ active phase. Blood was extracted by cardiac puncture with a 27-gauge needle and collected in an EDTA/heparin tube. Immediately after blood sampling, animals were transcardially perfused with a cold saline solution (0.9% NaCl). Perfusion was performed at a rate of 10 ml/min for 2–3 min per animal, corresponding to 20–30 ml. Perfusion was continued until 30 s after the perfusion fluid became completely transparent. Brain tissue from the prefrontal cortex, motor cortex and hippocampus were micro-dissected following the technique described by Chiu et al. (2007)^48^. The brain was removed from the skull and rinsed with ice-cold phosphate-buffered solution (PBS) to remove any surface blood. It was then placed on an ice-cold Petri dish and cut in half with a razor blade to separate the right and left hemispheres. Details of the dissection can be found in the Supplementary methods - dissection (item 1).

The dissected tissue was immediately treated with RNAlater™ according to the manufacturer’s instructions and subsequently stored at −20℃. The Gothenburg Ethical Committee for Animal Research approved all experiments (#181–2015), which were performed under relevant local and national guidelines and regulations. Additionally, the experiments were conducted following the ARRIVE guidelines 2.0, presented in the Supplemental file – ARRIVE 2.0 author checklist.

### Quantitative reverse transcription polymerase chain reaction (RT-qPCR)

Total RNA was extracted from the prefrontal cortex, motor cortex, and hippocampus according to Chomczynski and Sacchi with minor modifications described previously^49,50^. Optical density (OD) measurements 260/280 nm via NanoDrop 1000 (Thermo Scientific, USA) were used for RNA quantification. We also checked the RNA quality by running each sample in gel electrophoresis, where all samples showed intact 18 S and 28 S RNA bands, which is indicative of good quality. cDNA was prepared from 250 ng of total RNA using the HighCapacity cDNA Reverse Transcription Kit (Applied Biosystems, Foster city, CA, USA). RT-qPCR with preformed TaqMan gene expression assays (Applied Biosystems, USA; Table 1) together with a QuantStudio™ 3 real-time PCR system (Applied Biosystems, Carlsbad, CA, USA), was used to quantify the gene transcripts, for further information see http://www.appliedbiosystems.com). For details about RT-qPCR quantification, see the previously published supplementary information^42^. All transcript levels were normalized to the expression of *Gapdh* and thus are arbitrary but linear for each transcript, and comparisons between treatment groups represent actual linear changes. We tested two housekeeping genes. For GAPDH, we observed 10.1% lower values (*p* = 0.0004) in the running group, while Cyclophilin A (assay number: Mm02342430_g1) had 13.0% (*p* = 0.0002) lower values in the running group. We therefore chose to use only GAPDH for normalization (results for Cyclophilin A are not shown any further). Transcripts were grouped into four categories Hbb-like (*Hbb*,* Alas2*,* Alox15*), neuron-related (*Bdnf*,* Bax*,* Grin2a*,* Grin2b*), glia-related (*Hif1a*,* Gfap*), and IGF-1-related (*Igf1*,* Igf1r*,* Insr*,* Ghr*), as found in Table 1.

**Table 1:** Information on transcripts.

Abbreviation in Ms.	Assay number	Fullname	Category	Main function
*Hbb*	Mm01611268_g1	Hemoglobin, beta adult major chain	Hbb-like	Neuroprotection
*Alas2*	Mm00802083_m1	5’-aminolevulinate synthase 2	Hbb-like	Rate-controlling enzyme of heme biosynthesis (for functional hemoglobin assembly)
*Alox15*	Mm00507789_m1	Arachidonate 15-lipoxygenase	Hbb-like	Lipid peroxidating enzyme, cell membrane stability, memory, knock-in reduces hemoglobin
*Bdnf*	Mm04230607_s1	Brain-derived neurotrophic factor	Neuron	Regulator of synaptic transmission and plasticity
*Bax*	Mm00443039_m1	BCL2-associated X protein, apoptosis regulator	Neuron	Apoptosis regulator
*Grin2a*	Mm00433802_m1	Glutamate receptor, ionotropic, 2a	Neuron	Brain plasticity
*Grin2b*	Mm00433820_m1	Glutamate receptor, ionotropic, 2a	Neuron	Brain plasticity
*Hif1a*	Mm00468869_m1	Hypoxia-inducible factor 1. alpha subunit	Glia	Hypoxia-induced signaling protein
*Gfap*	Mm01253033_m1	Glial fibrillary acidic protein	Glia	Structural protein / morphogenesis
*Igf1*	Mm00439560_m1	Insulin-like growth factor 1	IGF-I-related	Brain plasticity
*Igf1r*	Mm00802831_m1	Insulin-like growth factor 1 receptor	IGF-I-related	Brain plasticity
*Insr*	Mm01211875_m1	Insulin receptor	IGF-I-related	Glucose metabolism/brain plasticity
*Ghr*	Mm00439093_m1	Growth hormone receptor	IGF-I-related	Brain plasticity
*Gapdh*	Mm99999915_g1	Glyceraldehyde-3-phosphate dehydrogenase	Reference gene	N/A

Main function for the transcripts, gene names and abbreviations of commercially available probes. The probes are assay-on-demand mixes of primers and TaqMan MGM probes (FAM dye labeled). References for the main function description are found in Supplementary Table S1 (ST1) (item 2). Further details can be obtained at http:/www.appliedbiosystems.com.

### Plasma analysis

The biochemical analysis for glucose, collected during the saline perfusion, was quantified using a glucose colorimetric assay kit, ab65333 – Glucose Assay kit, (Abcam, Cambridge, UK), according to the manufacturer’s instruction. In our experiments, the inter-assay variability was 3.8%. C-peptide quantification was performed using a sandwich-type immunoassay ELISA kit, Cat# 80-CPTMS-E01, (Alpco, Salem, NH, U.S.A), according to the manufacturer’s instruction. In our experiments, the inter-assay variability is 1.8%, and according to the manufacturer, the intra-assay variability is 4.1%. Insulin quantification was performed using a mouse insulin ELISA kit, Cat# EMINS, (Thermo Fisher Scientific, Carlsbad, CA 92008, U.S.A), according to the manufacturer’s instruction. The inter-assay variability was 8.7%, and according to the manufacturer, the intra-assay variability was < 10%. IGF-1 quantification was performed using an immunoassay solid phase ELISA, Cat# MG100 (R&D Systems, Inc, Minneapolis, MN 55413, U.S.A), according to the manufacturer’s instruction. The inter-assay variability was 5.4%, and according to the manufacturer, the intra-assay variability was 4.1%.

Homeostatic model of assessment of insulin resistance (HOMA-IR).

HOMA-IR was calculated by multiplying fasting insulin in IU/mL by fasting glucose in mmol/L, the sum divided by a factor of 22.5 ^51^.

### Statistical analysis

Values are presented as the mean ± 95% confidence interval (CI). Two-tailed *t*-test analyses were used for plasma and transcript-specific analyses for each brain region. The RT-qPCR gene expression values were logarithmically transformed to better approximate normal distribution, where appropriate. Apart from the individual *t*-tests for each brain region and transcripts, a mixed model was used to evaluate the aggregated effects of running concerning all brain regions and transcripts categories. Specifically, mixed models allow examining both fixed effects (as in the example of ordinary ANOVA) and random effects. Mouse was used as a random effect to account for the within-mouse correlation. We used maximum likelihood to deal with variations in data. Contrasts were constructed to compare the different brain regions and categories of transcripts rather than including category as a factor in the model. Spearman correlations were used to evaluate correlations, and rho (r), n, and p-values were presented. Correlation strengths (rho values; r) were reported according to Cohen^52^, being very small for *r* < 0.1, small for *r* < 0.1 to 0.3; moderate for *r* < 0.3 to 0.5, and large for *r* < 0.5. P values < 0.05 were considered statistically significant. SPSS (version 29.0.1.1) was used for statistical analyses.

For the running distance data, four out of twelve animals had partially missing data due to connectivity issues with the wireless running wheel, with recordings absent for two to five days. The missing values are presented in two ways (Supplementary Fig. 1 (item 3)). They were either set to 0 (without interpolation), or secondly, they were replaced with the average running distance for the dark or light cycle (with interpolation). Specifically, if a value was missing in the dark cycle, it was replaced by the average of all recorded dark cycle values for the same animal. Conversely, if a value was missing in the light cycle, it was replaced by the average of all recorded light cycle values for that animal.

## Results

### Running affects systemic and metabolic parameters

The running mice’s body weight tended to be slightly higher than that of the sedentary group (28.4 g vs. 27.1 g, *p* = 0.09) at the start of the experiment, which involved one week of non-running acclimatization with a locked running wheel. The average daily running distance (mean and 95% CI) was 3.07 ± 1.67 km/day; see Supplementary Fig. 1 (item 3); interpolated data, see Methods). The average percentage of time running during the day (mean and 95% CI) was 47.7 ± 12.3% and the average percentage of running activity during the active phase (mean and 95% CI) was 83.8 ± 4.3%. Running activity was not recorded in the sedentary group because the running wheel was locked. In Table 2, the measures reflecting systemic metabolism are presented. There were no differences between the groups regarding C-peptide, glucose, insulin plasma levels, or HOMA-IR index. For IGF-1, there was a tendency towards a slightly higher plasma level in the running group (+ 16%, *p* = 0.09; Table 2).

**Table 2:** Metabolic parameters.

	Unit	Sedentary	Runner	
		Mean ± 95% CI	Mean ± 95% CI	p-value
C-peptide	pmol/L	285 ± 32	262 ± 31	0.33
Glucose	mmol/L	5.71 ± 0.59	5.88 ± 0.41	0.63
Insulin	mU/L	1.65 ± 0.91	1.21 ± 0.32	0.39
IGF-1	ng/mL	172 ± 23	200 ± 21	0.09
HOMA - IR	Insulin x Glucose / 22.5	0.44 ± 0.26	0.33 ± 0.087	0.43

Abbreviations are homeostatic model of assessment of insulin resistance for HOMA-IR and insulin-like growth factor 1 for IGF-1. P-values are derived from T-tests. C-peptide (*n* = 11 for sedentary and *n* = 11 for runner), Glucose (*n* = 10 for sedentary and *n* = 11 for runner), Insulin (*n* = 9 for sedentary and *n* = 9 for runner) and IGF-1 (*n* = 11 for sedentary and *n* = 11 for runner). There was the same missing plasma sample in the running and sedentary group for all four parameters. For Glucose there was one hemolytic sample excluded whereas for Insulin, the additional missing values in the sedentary and running groups were due to levels in the range of blank (*n* = 2) or above the standard curve (*n* = 2).

Running increases Hbb-related transcripts and decreases IGF-1-related transcripts.

**Fig. 1:**
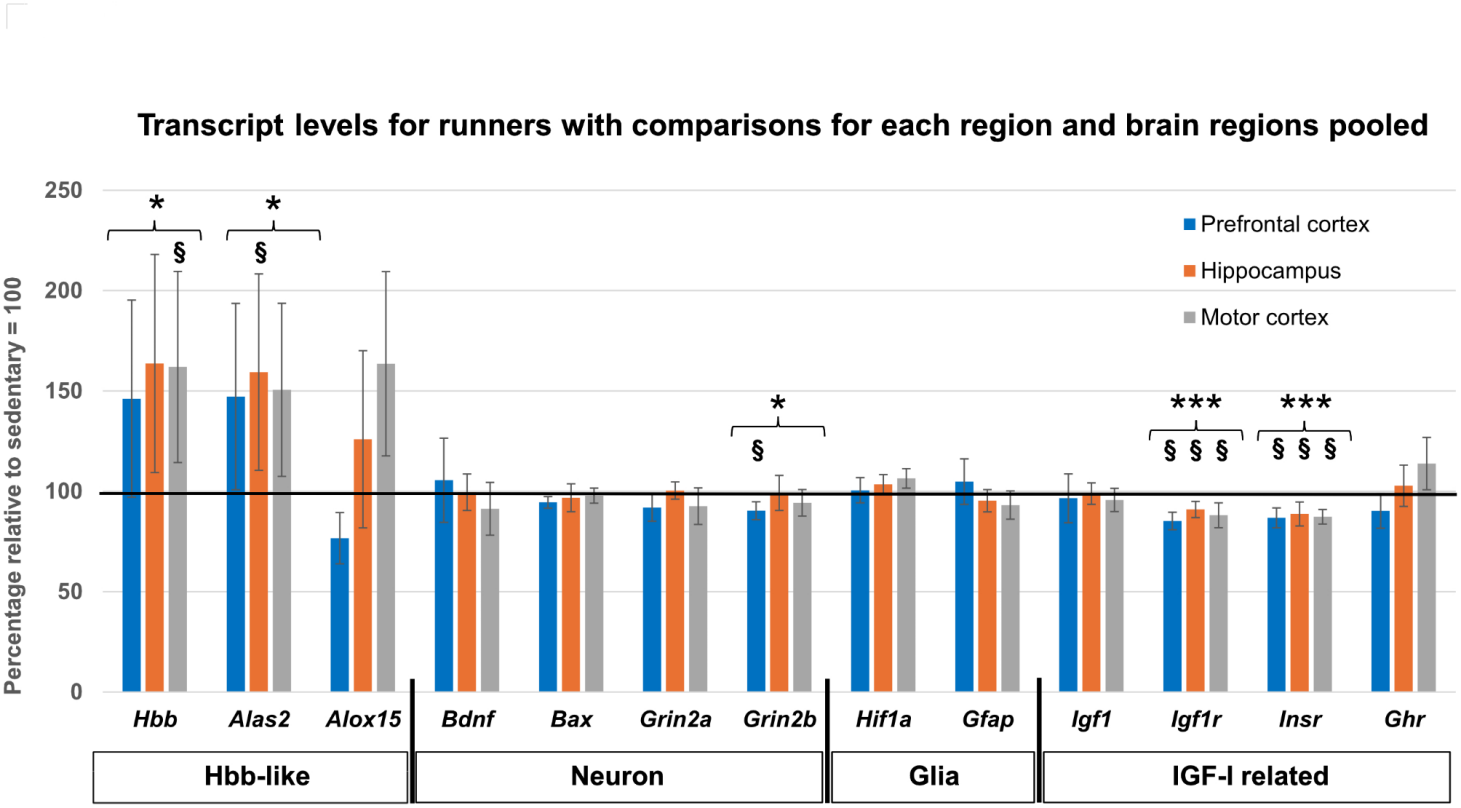
This Figure visually shows the levels of each transcript also shown in Tables 3 to facilitate comprehension of the pattern of changes in the specific brain regions and aggregates of the three brain regions. Total transcript levels for all three brain regions (prefrontal cortex, hippocampus, and motor cortex) are shown for runners as percentage vs. sedentary animals. Transcript levels have been normalized to Gapdh. Sedentary levels = 100%. Data is presented as mean ± 95% Cl. Individual transcript comparisons in each brain region are made with *t*-tests, and aggregated effects (brackets) in the three brain regions are made with mixed models (see Methods). *P < 0.05, ***P < 0.001, or *t*-test, ^§^*P* < 0.05, were used for statistical comparison as described in Methods.

The transcripts for *Hbb* and *Alas2* were upregulated after running in all three brain regions: prefrontal cortex, motor cortex, and hippocampus (Fig. 1; Table 3). The transcripts of the *Igf1r* and *Insr* were slightly downregulated (10–15%) by running in all three brain regions investigated. In addition, there was a slight decrease in the transcript *Grin2b* but only in the prefrontal cortex. The response to running was non-significant for the other transcripts investigated in the three brain regions examined.

**Fig. 2:**
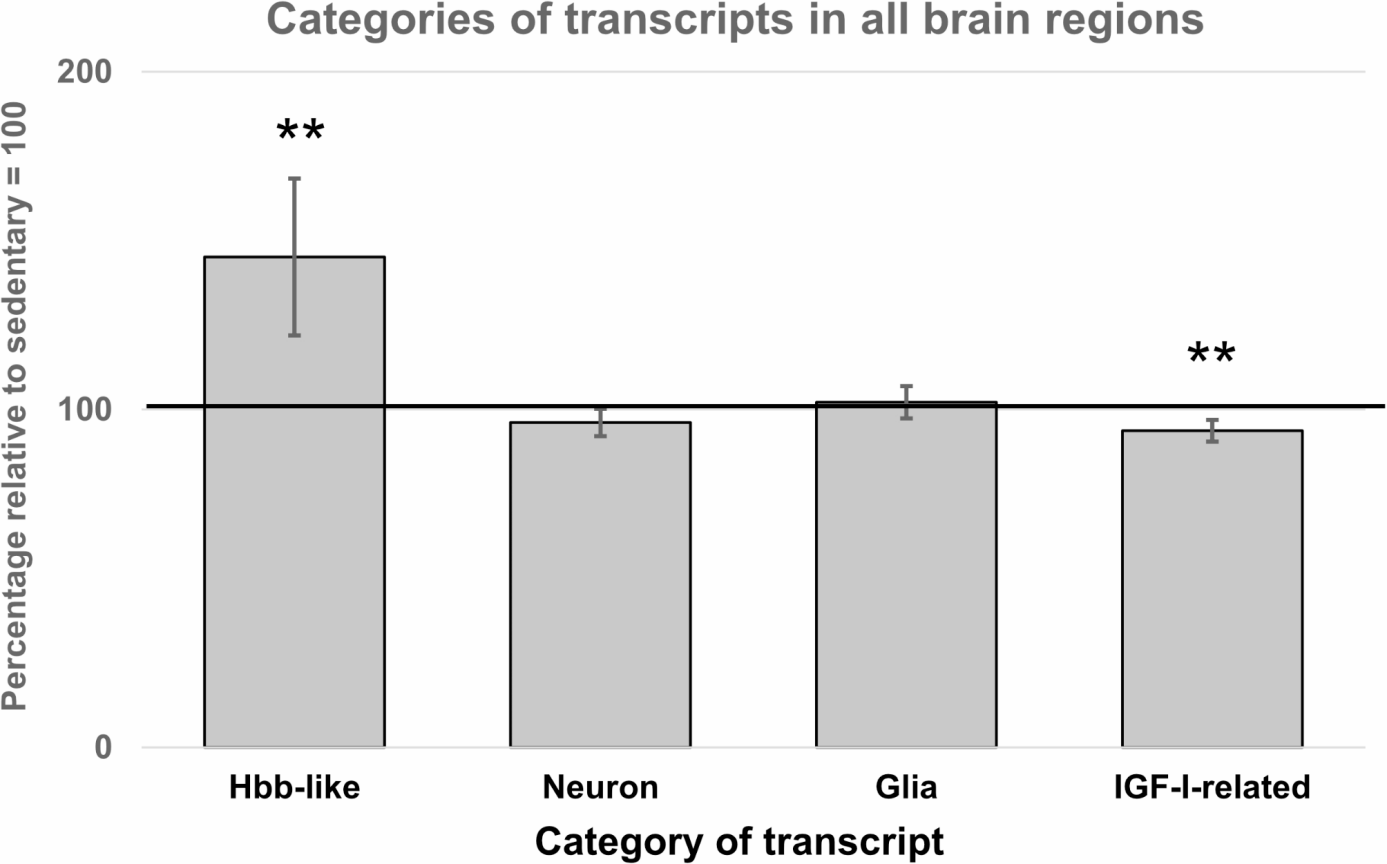
Levels of categories of transcripts in all brain regions (prefrontal cortex, hippocampus, and motor cortex). Aggregated comparisons are made with mixed model analysis, as described in Methods (statistical analysis). Sedentary levels = 100%. Data are presented as means ± 95% confidence intervals, **P < 0.01.

These patterns became even more apparent when the three brain regions were aggregated into one group (Fig. 2). Thus, these analyses confirmed that the running mice had significantly higher transcript levels in the Hbb-like category (45%) and significantly lower transcript levels in the IGF-1-related category (7%) (Fig. 2).

**Table 3:** Quantitative reverse transcription polymerase chain reaction (RT-qPCR) values in runners and sedentary animals.

Transcript	Group	Prefrontal Cortex	Hippocampus	Motor Cortex	Aggregated#
		Mean (95% CI)	Mean (95% CI)	Mean (95% CI)	Mean (95% CI)
*Hbb*	Runner	146.2 (97.1–195.3)	163.7 (109.3–218.0)	**161.9 (114.3–209.5)**	**157.2 (117.3–197.2)**
	Sedentary	100.0 (71.1–128.9)	100.0 (69.8–130.2)	100.0 (71.1–128.9)	100.0 (60.0–140.0)
*Alas2*	Runner	147.2 (100.8–193.6)	**159.4 (110.4–208.3)**	150.6 (107.4–193.7)	**152.4 (116.0–188.8)**
	Sedentary	100.0 (72.0–128.0)	100.0 (73.7–126.3)	100.0 (73.0–127.0)	100.0 (63.6–136.4)
*Alox15*	Runner	76.6 (63.9–89.4)	125.9 (81.8–170.1)	163.5 (117.6–209.5)	123.0 (95.8–150.2)
	Sedentary	100.0 (66.3–133.7)	100.0 (60.3–139.7)	100.0 (43.3–156.7)	99.7 (72.6–126.8)^£^
*BDNF*	Runner	105.5 (84.5–126.5)	99.5 (90.4–108.6)	91.3 (78.1–104.5)	98.8 (87.9–109.7)
	Sedentary	100.0 (89.0–111.0)	100.0 (89.6–110.4)	100.0 (88.7–111.3)	100.0 (89.1–110.9)
*Bax*	Runner	94.5 (91.6–97.4)	96.8 (89.9–103.7)	97.8 (94.1–101.6)	96.4 (93.3–99.5)
	Sedentary	100.0 (95.0–105.0)	100.0 (94.5–105.5)	100.0 (96.4–103.6)	100.0 (96.9–103.1)
*Grin2a*	Runner	91.9 (85.1–98.7)	100.4 (96.1–104.7)	92.6 (83.5–101.7)	95.0 (91.0–98.9)
	Sedentary	100.0 (91.1–108.9)	100.0 (95.4–104.6)	100.0 (93.5–106.5)	100.0 (96.1–103.9)
*Grin2b*	Runner	**90.3 (85.9–94.8)**	99.2 (90.4–107.9)	94.3 (87.6–100.9)	**94.6 (90.9–98.3)**
	Sedentary	100.0 (93.6–106.4)	100.0 (96.0–104.0)	100.0 (94.9–105.1)	100.0 (96.3–103.7)
*Hif1a*	Runner	100.5 (94.2–106.8)	103.5 (98.7–108.3)	106.4 (101.6–111.3)	103.5 (99.4–107.5)
	Sedentary	100.0 (94.4–105.6)	100.0 (94.8–105.2)	100.0 (95.5–104.5)	100.0 (96.0–104.0)
*Gfap*	Runner	104.8 (93.5–116.2)	95.3 (89.8–100.9)	93.2 (86.1–100.2)	100.8 (93.2–108.5)
	Sedentary	100.0 (85.5–114.5)	100.0 (91.0–109.1)	100.0 (85.5–114.5)	100.0 (92.4–107.6)
*Igf1*	Runner	96.5 (84.4–108.6)	98.8 (93.5–104.2)	95.7 (89.9–101.5)	97.0 (92.1–101.9)
	Sedentary	100.0 (94.1–105.9)	100.0 (95.6–104.4)	100.0 (94.4–105.6)	100.0 (95.1–104.9)
*Igf1r*	Runner	**85.2 (80.9–89.6)**	**90.9 (86.9–94.9)**	**88.1 (81.9–94.2)**	**88.1 (84.8–91.3)**
	Sedentary	100.0 (95.5–104.5)	100.0 (95.1–104.9)	100.0 (95.3–104.7)	100.0 (96.8–103.2)
*Insr*	Runner	**86.8 (81.8–91.7)**	**88.7 (82.8–94.7)**	**87.3 (83.7–91.0)**	**87.6 (83.4–91.8)**
	Sedentary	100.0 (94.9–105.1)	100.0 (93.1–106.9)	100.0 (94.9–105.1)	100.0 (95.8–104.2)
*Ghr*	Runner	90.2 (81.6–98.8)	102.8 (92.5–113.1)	113.8 (100.8–126.8)	102.3 (95.1–109.4)
	Sedentary	100.0 (92.9–107.1)	100.0 (92.8–107.2)	100.0 (90.1–109.9)	100.0 (92.9–107.1)

The values are normalized against sedentary = 100. Mean and variation is given as 95% confidence intervals (95% CI). Significant differences (*p* < 0.05) are in bold and designate *t*-test comparisons between runners and sedentary for each brain region. #For all three brain regions aggregated, the mixed model was used for statistical comparison between sedentary and runner (see Methods). The average sedentary values for each brain region were normalized to 100. ^£^The reason for that sedentary was 99.7 for *Alox15* in the three brain regions were some missing values (motor cortex; *n* = 2, prefrontal cortex; *n* = 1, hippocampus; *n* = 1). The mixed model algorithm considers this and balances missing values slightly, which slightly changed the calculated mean for this group. The other transcripts had no missing values, and adjusted sedentary means were all 100.0. There were however also missing values in the running group (prefrontal cortex; *n* = 3, hippocampus; *n* = 1), which also slightly changed the calculated means (but this is not as easily observed, as means were not expected to be 100). This is also graphically shown in Fig. 2. Transcript abbreviations are found in Table 1.

### Hbb-related gene expression correlates with the IGF-1 system components in running animals

To further indicate changes in relationships between runners and sedentary animals, we performed correlation analyses between Hbb-related transcripts and systemic and local brain components of the IGF-1 system (Table 4). In the running group, *Hbb* and *Alas2* moderately negatively correlated with both *Igf1* and *Ghr*, while *Alox15* had a moderate positive correlation with *Ghr*. In the sedentary group, there were no significant correlations between these transcripts (Table 4). For plasma glucose, there was a moderate negative correlation with *Igf1* and *Igf1r* in the running group. The sedentary group had a moderate negative correlation between glucose and *Insr*. In the running group, HOMA-IR showed a large negative correlation with *Igf1r* and a positive moderate correlation with *Insr* and *Ghr*. Still, there were no significant correlations in the sedentary group.

**Table 4:** Correlations between Hbb-related transcripts, plasma-IGF-I, glucose and HOMA-IR in running and sedentary mice.

Correlations		Runner				Sedentary			
(Spearman’s rho)		*Igf1*	*Igf1r*	*Insr*	*Ghr*	*Igf1*	*Igf1r*	*Insr*	*Ghr*
*Hbb*	rho	**−0.38**	−0.25	−0.074	**−0.33**	0.26	0.025	−0.19	0.037
	p	**0.023**	0.14	0.67	**0.048**	0.12	0.88	0.27	0.83
	N	36	36	36	36	36	36	36	36
*Alas2*	rho	**−0.35**	−0.27	−0.016	**−0.34**	0.33	0.047	−0.18	0.015
	p	**0.038**	0.11	0.93	**0.043**	0.051	0.79	0.29	0.93
	N	36	36	36	36	36	36	36	36
*Alox15*	rho	0.13	0.25	−0.095	**0.49**	−0.15	−0.23	−0.17	−0.005
	p	0.47	0.17	0.60	**0.004**	0.43	0.22	0.37	0.98
	N	33	33	33	33	32	32	32	32
Plasma-IGF1	rho	−0.16	−0.21	**−0.36**	**−0.50**	−0.12	**−0.35**	**-0.40**	0.20
	p	0.36	0.25	**0.042**	**0.003**	0.50	**0.049**	**0.022**	0.26
	N	33	33	33	33	33	33	33	33
Glucose	rho	**-0.35**	**-0.37**	0.31	−0.017	0.034	-0.10	**-0.41**	-0.018
	p	**0.044**	**0.036**	0.078	0.92	0.86	0.60	**0.024**	0.92
	N	33	33	33	33	30	30	30	30
HOMA-IR	rho	-0.33	**-0.53**	**0.46**	0.36	0.39	0.15	−0.14	−0.14
	p	0.094	**0.005**	**0.016**	0.067	0.060	0.48	0.50	0.52
	N	27	27	27	27	24	24	24	24

The Spearman correlations are between the values for all values of all brain regions versus the levels of each transcript. Abbreviations are as in Table 1. and HOMA-IR is the homeostatic model of assessment of insulin resistance. N represents the pooled individual samples with successful analysis for all three brain regions. The reasons for not reaching *N* = 36 (i.e. missing numbers) are given in Tables 2, 3.

## Discussion

The main finding of the present study is that exercise increased the expression of the *Hbb* and *Alas2* transcripts in three regions of the mouse brain. Additionally, running slightly decreased IGF-1-related transcripts, whereas systemic components were unaffected. Interestingly, the correlation analyses only yielded significant results in the running group and for Hbb-like transcripts (Table 4). However, a negative correlation was found between local brain *Insr* and glucose in the sedentary group. We have previously observed increased Hbb-like transcripts when Hx rodents received replacement with GH and IGF-1 ^41^. In the present study, the increase in these transcripts after running appears to be similar but of less magnitude to that seen after GH or IGF-1 replacement in Hx rodents. Furthermore, we found a tendency towards a 16% increase in peripheral plasma IGF-1 after running. This is in line with reports showing that effects of exercise on brain function are mediated by increased uptake of circulating IGF-1 ^4^, which is necessary for the generation of new hippocampal neurons^53^.

Regarding Hbb and Alas2 in the brain, it could be questioned whether the measured transcript levels represent their expression in the brain or originate from the bloodstream. We have previously shown^54^, that *Hbb* and *Alas2* detected in the brain at most have minor contributions (approximately 1–10%) from remaining blood cells. Therefore, the presently detected levels of *Hbb* and *Alas2* are indeed from the brain, although some minor contamination from the bloodstream cannot be ruled out.

We also detected that running decreased the expression of the *Insr* and *Igf1r* transcripts. Previously, it has been shown that expression of *Insr* decreases in running vs. sedentary rats^55^. Furthermore, another study reports that insulin transport across the blood-brain barrier is augmented following exercise, although serum insulin levels are unchanged^56^. Moreover, higher IGF-1 in plasma may have produced a lowering effect on the expression of *Igf-1r* potentially induced by negative feedback. In contrast, our study could not detect any difference in plasma insulin levels, glucose levels, or HOMA-IR index between the running and sedentary mice. Body weight was similar in the two groups, which suggests that metabolism was minimally affected by the 7-day running paradigm.

When selecting 6-month-old mice for this study, we aimed to achieve a high capacity for voluntary running to capture an exercise-induced upregulation of neuronal Hbb. Total voluntary running distance has been reported to decline by approximately 45% in mice around 10 months old compared to younger mice aged 5 to 14 weeks^57^. However, these animals are relatively healthy in terms of metabolism. The effects of endurance exercise on metabolic profile would likely have been more apparent in older or obese animals with insulin resistance. Also, other breeds of mice with a genetic risk of insulin resistance, such as GLUT4 +/- mice^58,59^ and db/db mice^60^, could have been used to find more robust effects of running on insulin resistance. The generalizability is probably high to other rodent strains, but somewhat less known to humans. However, in general, exercise and insulin resistance have rather similar relations in humans and rodents, with larger variations for use of different ages and comorbidities.

We identified that both *Hbb* and *Alas2* correlated with *Igf1* and *Ghr* in the running group, which further strengthens our hypothesis that there is an association between Hbb-like and IGF-1-related transcripts. While *Alox15* was not significantly increased in any of the specific brain regions, there was a numerical increase (Table 3), which in the aggregated mixed model analysis for the three brain regions and *Hbb*, *Alas2*, and *Alox15* became significant (Fig. 2). Although *Alox15* has some relation with *Hbb* and *Alas2* in terms of affecting hemoglobin levels^40^, and in terms of being increased by GH treatment in parallel with the equally increased *Hbb* and *Alas2*^41^, its direct function in synthesizing lipid metabolites is nevertheless unrelated to hemoglobin synthesis. Therefore, it may not be surprising that *Alox15* is less affected by running than *Hbb* and *Alas2*. Also, running in the present experiment increased plasma IGF-1 (16%, *p* = 0.09) less than in the GH treatment mentioned (≈ 2.5-fold^41^).

Further on, neither neuro-related nor glia-related category transcripts were affected by running exercise. Apart from direct effects on the levels of transcripts by running, correlation patterns may indicate the presence of actively functioning pathways, for example, through IGF1 stimulation of the IGF-1R. Interestingly, significant moderate to large correlations were found almost exclusively in the running group (Table 4), which further supports the notion that there is a relation between Hbb-like transcripts and the local IGF-1 system in the brain, which is activated in runners. In the literature, we could not find any evidence that an association between Hbb and IGF-1 in the brain has previously been shown. Also, it appears that some of the small (*r* = 0.1–0.3) non-significant correlations may have some biological significance but would have required higher statistical power, i.e., more animals, to have been detected significant. Also, it deserves to be mentioned that correlation patterns should be interpreted with caution and should not be regarded as causal.

Our results suggest that running may indeed increase brain Hbb-like transcripts. This presents a new mechanism of action for the previously known neuroprotective resilience of running^61–63^. Therefore, Hbb-like transcripts and proteins may be a target of intervention to convey neuroprotection in brain ischemia. This could be investigated in experimental stroke studies, perhaps using running intervention. Further, it would be interesting to check the single-cell expression of Hbb in different brain cell types and to do a knockout model of Hbb, specifically in neurons.

## Summary

Running for seven days substantially increased Hbb-related transcripts in three brain regions. This may be due to an increase in peripheral IGF-1 and an increased transport of insulin into the brain after exercise, which in turn could contribute to a lowering of the local *Insr* and *Igf1r* transcripts. The correlations between Hbb-like and IGF-1 system transcripts in the running group but not in the sedentary group further support a link between IGF-1-related and Hbb-like transcripts in response to exercise.

## Electronic supplementary material

Below is the link to the electronic supplementary material.

Supplementary Material 1

Supplementary Material 2

## Data Availability

The datasets generated during and/or analyzed during the current study are available from the corresponding author on reasonable request.
