# Supplementary material for: Running in mice increases the expression of brain hemoglobin-related genes interacting with the GH/IGF-1 system
Source: Sci Rep. 2024 Oct 26;14:25464. doi: 10.1038/s41598-024-77489-1 (PMC11513053; doi:10.1038/s41598-024-77489-1)
Supplement: Supplementary file 2 — Supplementary Material 2 [file 41598_2024_77489_MOESM2_ESM.docx]

#

# **Running in mice increases the expression of brain hemoglobin-related genes interacting with the GH/IGF-1 system**

**Authors:**

Marion Walser^1,2^**^*^**, Lars Karlsson^3,4^, Reza Motalleb^3^, Jörgen Isgaard^1,6^, H Georg Kuhn^3,5^, Johan Svensson^1^, N. David Åberg^1^.

**Supplementary Material (items)**

1. Supplementary methods - dissection
2. Supplementary Table S1 (ST1)
3. Supplementary Figure 1 (SF1)
4. Supplementary references
5. **Supplementary methods – dissection:**

Brain tissue from the prefrontal cortex, motor cortex and hippocampus were micro-dissected following the technique described by Chiu et al. (2007) ^1^ as also stated in the main text. Here follows a more detailed description of the micro-dissection performed.

The following razor blade cuts were made using a dissecting microscope. The first coronal cut separated the olfactory bulb at the border of the anterior part of the isocortex. The second coronal cut was made at the anterior point of the corpus callosum, from which the isocortex was separated from the underlying corpus callosum, caudate putamen, and anterior olfactory nucleus, and collected as the prefrontal cortex. The third coronal cut was made at the ventral point of the fornix, separating the isocortex from the underlying corpus callosum, and collected as the motor cortex. Lastly, the hemispheres were positioned with the ventral sides up to remove the midbrain and expose the hippocampi. The hippocampi were collected in full by gently rolling them out using a spatula.

1. **Supplementary Table S1**

| **Supplementary Table S1.** Key references of examined transcripts. | |  |  |
| --- | --- | --- | --- |
| **Fullname and gene symbol** | **Category#** | **Main function#** | **Reference(s)** |
| Hemoglobin, beta adult major chain (Hbb-b1) | Hbb-like | neuroprotection | 2-7-6 |
| 5'-aminolevulinate synthase 2 (Alas2) | Hbb-like | rate-controlling enzyme of heme biosynthesis (for functional hemoglobin assembly) | 8,9 |
| Arachidonate 15-lipoxygenase (Alox15) | Hbb-like | lipid peroxidating enzyme, cell membrane stability, memory,knock-in reduces hemoglobin. | 4,10-12 |
| Brain-derived neurotrophic factor (Bdnf) | Neuron | regulator of synaptic transmission and plasticity | 13 |
| BCL2-associated X protein, apoptosis regulator (Bax) | Neuron | apoptosis regulator | 14,15 |
| Glutamate receptor, ionotropic, 2a (Grin2a) | Neuron | brain plasticity | 16-18 |
| Glutamate receptor, ionotropic, 2a (Grin2b) | Neuron | brain plasticity | 16-18 |
| Hypoxia-inducible factor 1. alpha subunit (Hif1a) | Glia | hypoxia-induced signaling protein | 19 |
| Glial fibrillary acidic protein (Gfap) | Glia | structural protein / morphogenesis | 20 |
| Insulin-like growth factor 1 (Igf1) | IGF-I-related | brain plasticity | 21-26 |
| Insulin-like growth factor 1 receptor (Igf1r) | IGF-I-related | brain plasticity | 17 |
| Insulin receptor (Insr) | IGF-I-related | glucose metabolism/brain plasticity | 27 |
| Growth hormone receptor (Ghr) | IGF-I-related | brain plasticity | 16 |
| Glyceraldehyde-3-phosphate dehydrogenase (Gapdh) | reference gene | N/A |  |
| **Footnote:** #This information is also given in the main text but is additionally shown here for clarity. Although some of these references are found in the main text, this is a considerably expanded reference list, which is the reason for having a separate numbering as compared to the main text. | | | |

1. **Supplementary Figure 1.**


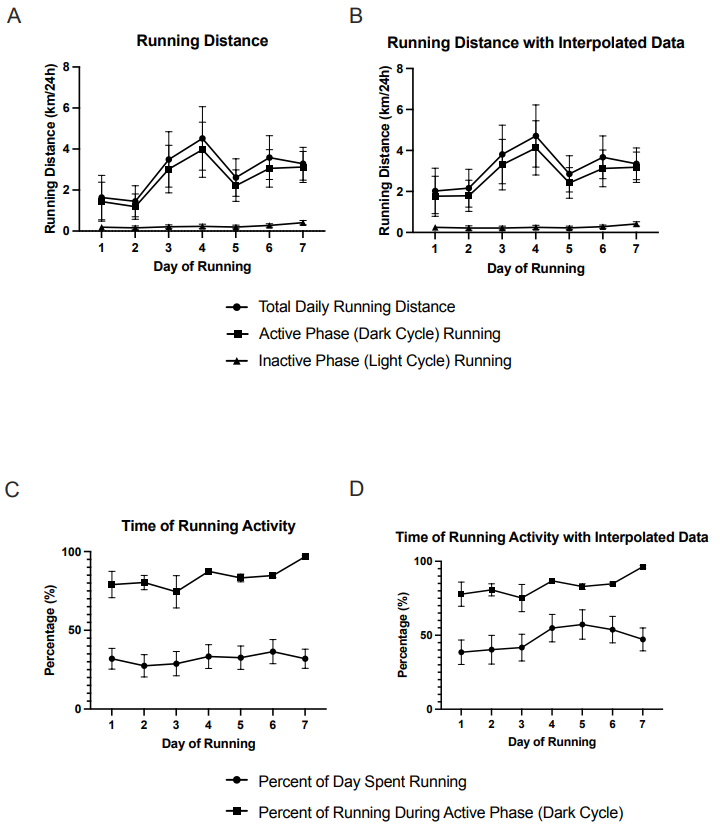


**Figure Legend: Running Distance and Running Activity with and without Interpolation of Missing Data Values****.** When collecting data, it was discovered that the telemetry system did not signal the data properly for some mice (n=4 out of the full n=12) for 2-5 days. Missing data was handled in two ways as described in Methods, statistical analysis (being set to 0 [without interpolation], or being replaced by the average of recorded data [interpolation]. Image (A) shows the average daily running distance along with the average distance per day for the dark cycles (active phase) and the light cycles (inactive phase) without interpolation. Image (B) shows the average running distances each day, dark and light cycles with interpolation. Image (C) shows the percentage of time animals spent running each day and the percentage of total distance the animals ran during the active phase (dark cycle) without interpolating missing data values. Image (D) shows the percentages of time and distance in dark cycle with interpolating missing data values. Data is presented as mean ± standard error of the mean (SEM).

1. **Supplementary references:**

A few of these references are also referenced in the main text.

1. Chiu, K., Lau, W. M., Lau, H. T., So, K. F. & Chang, R. C. Micro-dissection of rat brain for RNA or protein extraction from specific brain region. *Journal of visualized experiments : JoVE*, 269 (2007). [https://doi.org:10.3791/269](about:blank)
2. Ohyagi, Y., Yamada, T. & Goto, I. Hemoglobin as a novel protein developmentally regulated in neurons. *Brain Res* **635**, 323-327 (1994).
3. He, Y. *et al.* Brain Alpha- and Beta-Globin Expression after Intracerebral Hemorrhage. *Translational Stroke Research* **1**, 48-56 (2010). [https://doi.org:10.1007/s12975-009-0004-x](about:blank)
4. Yan, H. *et al.* Circulating IGF1 regulates hippocampal IGF1 levels and brain gene expression during adolescence. *J Endocrinol* **211**, 27-37 (2011). https://doi.org:10.1530/joe-11-0200
5. Walser, M. *et al.* Peripheral administration of bovine GH regulates the expression of cerebrocortical beta-globin, GABAB receptor 1, and the Lissencephaly-1 protein (LIS-1) in adult hypophysectomized rats. *Growth Horm Igf Res* **21**, 16-24 (2011). [https://doi.org:10.1016/j.ghir.2010.11.002](about:blank)
6. Walser, M. *et al.* Different modes of GH administration influence gene expression in the male rat brain. *J Endocrinol* **222**, 181-190 (2014). https://doi.org:10.1530/JOE-14-0223
7. Walser, M. *et al.* Mode of GH administration and gene expression in the female rat brain. *J Endocrinol* **233**, 187-196 (2017). [https://doi.org:10.1530/joe-16-0656](about:blank)
8. Sadlon, T. J., Dell'Oso, T., Surinya, K. H. & May, B. K. Regulation of erythroid 5-aminolevulinate synthase expression during erythropoiesis. *Int J Biochem Cell Biol* **31**, 1153-1167 (1999).
9. Zhang, F. L. *et al.* Hypoxic induction of human erythroid-specific delta-aminolevulinate synthase mediated by hypoxia-inducible factor 1. *Biochemistry* **50**, 1194-1202 (2011). [https://doi.org:10.1021/bi101585c](about:blank)
10. Schäfer, M. *et al.* Male Knock-in Mice Expressing an Arachidonic Acid Lipoxygenase 15B (Alox15B) with Humanized Reaction Specificity Are Prematurely Growth Arrested When Aging. *Biomedicines* **10** (2022). https://doi.org:10.3390/biomedicines10061379
11. Shalini, S. M. *et al.* Distribution of Alox15 in the Rat Brain and Its Role in Prefrontal Cortical Resolvin D1 Formation and Spatial Working Memory. *Mol Neurobiol* **55**, 1537-1550 (2018). [https://doi.org:10.1007/s12035-017-0413-x](about:blank)
12. Wittwer, J. & Hersberger, M. The two faces of the 15-lipoxygenase in atherosclerosis. *Prostaglandins, leukotrienes, and essential fatty acids* **77**, 67-77 (2007). https://doi.org:10.1016/j.plefa.2007.08.001
13. Rasmussen, P. *et al.* Evidence for a release of brain-derived neurotrophic factor from the brain during exercise. *Experimental physiology* **94**, 1062-1069 (2009). [https://doi.org:10.1113/expphysiol.2009.048512](about:blank)
14. Aboutaleb, N. *et al.* Protection of Hippocampal CA1 Neurons Against Ischemia/Reperfusion Injury by Exercise Preconditioning via Modulation of Bax/Bcl-2 Ratio and Prevention of Caspase-3 Activation. *Basic Clin Neurosci* **7**, 21-29 (2016).
15. Dehqanizadeh, B., Mohammadi, Z. F., Kalani, A. H. T. & Mirghani, S. J. Effect of early exercise on inflammatory parameters and apoptosis in CA1 area of the hippocampus following cerebral ischemia-reperfusion in rats. *Brain Res Bull* **182**, 102-110 (2022). [https://doi.org:10.1016/j.brainresbull.2022.02.011](about:blank)
16. Le Greves, M., Steensland, P., Le Greves, P. & Nyberg, F. Growth hormone induces age-dependent alteration in the expression of hippocampal growth hormone receptor and N-methyl-D-aspartate receptor subunits gene transcripts in male rats. *Proc Natl Acad Sci U S A* **99**, 7119-7123 (2002). [https://doi.org:10.1073/pnas.092135399](about:blank) 99/10/7119 [pii]
17. Le Greves, M. *et al.* Growth hormone replacement in hypophysectomized rats affects spatial performance and hippocampal levels of NMDA receptor subunit and PSD-95 gene transcript levels. *Exp Brain Res* **173**, 267-273 (2006). [https://doi.org:10.1007/s00221-006-0438-2](about:blank)
18. Molina, D. P., Ariwodola, O. J., Weiner, J. L., Brunso-Bechtold, J. K. & Adams, M. M. Growth hormone and insulin-like growth factor-I alter hippocampal excitatory synaptic transmission in young and old rats. *Age*, 1-13 (2012).
19. Nair, D., Ramesh, V., Li, R. C., Schally, A. V. & Gozal, D. Growth hormone releasing hormone (GHRH) signaling modulates intermittent hypoxia-induced oxidative stress and cognitive deficits in mouse. *Journal of neurochemistry* **127**, 531-540 (2013). [https://doi.org:10.1111/jnc.12360](about:blank)
20. Pekny, M. *et al.* Mice lacking glial fibrillary acidic protein display astrocytes devoid of intermediate filaments but develop and reproduce normally. *EMBO J* **14**, 1590-1598 (1995).
21. Lopez-Fernandez, J. *et al.* Growth hormone induces somatostatin and insulin-like growth factor I gene expression in the cerebral hemispheres of aging rats. *Endocrinology* **137**, 4384-4391 (1996).
22. Ye, P. *et al.* Regulation of insulin-like growth factor I (IGF-I) gene expression in brain of transgenic mice expressing an IGF-I-luciferase fusion gene. *Endocrinology* **138**, 5466-5475 (1997).
23. Adams, M. M. *et al.* Stability of local brain levels of insulin-like growth factor-I in two well-characterized models of decreased plasma IGF-I. *Growth Factors* **27**, 181-188 (2009). [https://doi.org:910188605](about:blank) [pii] 10.1080/08977190902863639
24. Ding, Q., Vaynman, S., Akhavan, M., Ying, Z. & Gomez-Pinilla, F. Insulin-like growth factor I interfaces with brain-derived neurotrophic factor-mediated synaptic plasticity to modulate aspects of exercise-induced cognitive function. *Neuroscience* **140**, 823-833 (2006). [https://doi.org:10.1016/j.neuroscience.2006.02.084](about:blank)
25. Llorens-Martín, M., Torres-Alemán, I. & Trejo, J. L. Mechanisms mediating brain plasticity: IGF1 and adult hippocampal neurogenesis. *The Neuroscientist : a review journal bringing neurobiology, neurology and psychiatry* **15**, 134-148 (2009). [https://doi.org:10.1177/1073858408331371](about:blank)
26. Ohlsson, C. *et al.* The role of liver-derived insulin-like growth factor-I. *Endocr Rev* **30**, 494-535 (2009). https://doi.org:10.1210/er.2009-0010
27. Brown, C. *et al.* Insulin blood-brain barrier transport and interactions are greater following exercise in mice. *Journal of applied physiology (Bethesda, Md. : 1985)* **132**, 824-834 (2022). [https://doi.org:10.1152/japplphysiol.00866.2021](about:blank)
